# Naringin Prevents Cognitive Dysfunction in Aging Rats by Inhibiting Toll-Like Receptor 4 (TLR4)/NF-*κ*B Pathway and Endoplasmic Reticulum Stress

**DOI:** 10.1155/2023/2919811

**Published:** 2023-02-21

**Authors:** Xiao-jie Dai, Yi Jia, Rui Cao, Mei-ning Zhou

**Affiliations:** ^1^Internal Medicine-Neurology, Xi'an Gaoxin Hospital, Shaanxi 710075, China; ^2^Internal Medicine-Neurology, Qingyang People's Hospital, Qingyang, Gansu 745000, China; ^3^The Third Affiliated Hospital of Xi'an Medical University, Xi'an, Shaanxi 710068, China

## Abstract

**Objective:**

Naringin is a flavonoid derived from Chinese herbs. According to earlier studies, naringin may have the potential to alleviate aging-induced cognitive dysfunction. Therefore, this study attempted to explore the protective effect and underlying mechanism of naringin on aging rats with cognitive dysfunction.

**Methods:**

After the construction of a model of aging rats with cognitive dysfunction through subcutaneous injection of D-galactose (D-gal; 150 mg/kg), intragastric administration of naringin (100 mg/kg) was performed for treatment. Behavioral tests, including Morris water maze test (MWM), novel object recognition test (NORT), and fear conditioning test, were used to measure the cognitive function; ELISA and biochemical tests were used to determine the levels of interleukin (IL)-1*β*, IL-6, monocyte chemoattractant protein-1 (MCP-1), brain-derived neurotrophic factor (BDNF), nerve growth factor (NGF), malondialdehyde (MDA), and glutathione peroxidase (GSH-Px) in the hippocampus of rats in each group, respectively; H&E staining was used to observe the pathological changes in the hippocampus; Western blot was used to examine the expression of toll-like receptor 4 (TLR4)/NF-*κ*B pathway-related proteins and endoplasmic reticulum (ER) stress-related proteins in the hippocampus.

**Results:**

The model was successfully constructed by subcutaneous injection of D-gal (150 mg/kg). The behavioral test results showed that naringin could ameliorate the cognitive dysfunction and alleviate the histopathological damage of hippocampus. Moreover, naringin significantly improve the inflammatory response (the levels of IL-1*β*, IL-6, and MCP-1 were decreased), oxidative stress response (MDA level was increased while GSH-Px activity was decreased), and ER stress (the expression of glucose-regulated protein 78 (GRP78), C/-EBP homologous protein (CHOP), and transcription factor 6 (ATF6) expression was downregulated), and increased the levels of neurotrophic factors BDNF and NGF in D-gal rats. Besides, further mechanistic studies revealed the downregulation of naringin on TLR4/NF-*κ*B pathway activity.

**Conclusion:**

Naringin may inhibit inflammatory response, oxidative stress, and ER stress by downregulating TLR4/NF-*κ*B pathway activity, thereby improving cognitive dysfunction and alleviating histopathological damage of hippocampus in aging rats. Briefly, naringin is an effective drug for the treatment of cognitive dysfunction.

## 1. Introduction

Despite a normal physiological process, aging raises the risk of diabetes, vasculopathy, hypertension, dyslipidemia, and dementia [1]. Individuals pay more and more attention to the aging issue with the increase in the aging population proportion and the number of aging countries. Studies have suggested that the old behave poorer than the young in memory, attention, visuospatial ability, or executive function [2]. The poor performance of the old is closely related to neurobiological changes in the hippocampus, such as a decrease in new neurons in the subgranular zone of the hippocampus [3]. And neurobiological changes in the hippocampus may be attributed to factors such as chronic inflammation, oxidative stress, and mitochondrial dysfunction. According to several types of research, the brain falls short of defense mechanisms such as inflammation, oxidative damage, and antioxidant in different regions during aging [4]. Also, studies have demonstrated that inflammatory markers, such as tumor necrosis factor-*α* (TNF-*α*) and interleukin (IL)-1*β*, are elevated in the old and associated with age-related cognitive dysfunction and decline [5]. In addition, aging-induced protein damage and changes in redox status result in the accumulation of proteins with decreased folding abilities and misfolded proteins in the endoplasmic reticulum (ER) cavity, thereby activating a series of signaling pathways. The above process is called ER stress, and generally, the accumulation of ER stress can result in apoptosis [6]. Decreased learning and memory abilities caused by the deterioration of brain function during the physiological process of aging are tightly related to the accumulation of inflammation, oxidative damage, and ER stress.

Traditional Chinese medicine (TCM), a system of traditional medicine in the Chinese healthcare system over thousands of years, plays a crucial role in the treatment of chronic diseases such as lung cancer, coronary heart disease, allergies, diabetes, and infections [7]. Naringin, a natural flavanone glycoside, is mainly present in Chinese herbs, tomatoes, and citrus fruits. Prior studies have stated that the malignant behavior of various cancers, including esophageal cancer [8], gastric cancer [9], colorectal cancer [10], and ovarian cancer [11], was inhibited by naringin. In addition, various biological and pharmacological properties of naringin, such as anti-inflammation, antioxidation, and antiapoptosis, have also been demonstrated by some studies [12–14]. Moreover, other research has revealed that naringin can effectively reduce the expression of inflammation-related factors. For example, Habauzit et al. claimed that naringin significantly inhibited the expression of proinflammatory cytokine IL-6 in the serum of aging Wistar rats [15]. Also, naringin has antioxidant abilities. Golechha et al. discovered that naringin inhibited oxidative stress in rats with pentylenetetrazole-induced epilepsy by binding to free radicals and regulating GSH levels [16]. Notably, Wang et al. discovered that oral naringin greatly improved learning and memory abilities and alleviated mitochondrial dysfunction in rats given a high-fat diet (HFD) for 20 weeks [17]. The above findings suggest that naringin has the potential to treat aging-induced cognitive dysfunction in old people, but there are few related studies at present. Therefore, based on the model of aging rats with cognitive dysfunction induced by subcutaneous injection of D-galactose (D-gal), the effect and possible mechanism of naringin on these rats were investigated through a series of behavioral tests, biochemical experiments, and other basic studies. And the objective of this paper was to propose novel perspectives for the prevention and improvement of aging-induced cognitive dysfunction.

## 2. Materials and Methods

### 2.1. Construction and Processing of the Rat Model

This study was approved by the Ethics Committee of The Third Affiliated Hospital of Xi'an Medical University (XAMU2022-05) and conducted under the approved guidelines. To explore the effect of naringin on cognitive dysfunction in aging rats, we constructed an aging rat model of cognitive dysfunction by subcutaneous injection of D-gal (150 mg/kg) following the method in the study by Garcez et al. [18]. Twenty Wistar rats (weight: 220 ± 20 g) were randomly divided into 4 groups (*n* = 5). In the control group, rats were injected subcutaneously and gavaged with an equal amount of saline. In the naringin gavage (NG) group, rats were injected subcutaneously with an equal amount of saline and gavaged with naringin (100 mg/kg/d, CAS NO. 10236-47-2, MedChemExpress, USA) for 8 weeks. In the D-gal group, after the construction of a model of aging rats with cognitive dysfunction through subcutaneous injection of D-gal (150 mg/kg), rats were treated with intragastric administration of an equal amount of saline. As for the D-gal + NG group, a model of rats with cognitive impairment was established by subcutaneous injection of D-gal (150 mg/kg), and then, rats were gavaged with naringin (100 mg/kg/d) for 8 weeks. At the end of the experimental cycle, the rats were subjected to the Morris water maze test (MWM) and novel object recognition test (NORT), followed by euthanasia. Finally, their hippocampal tissues were collected for subsequent tests.

### 2.2. Morris Water Maze Test

The MWM test was employed to assess the spatial learning and memory ability of rats in each group. MWM was a circular pool filled with water (21 ± 1°C), with a white inner wall, 150 cm diameter, and 50 cm height. The pool was divided into four quadrants labeled with different markers. After the last intervention, the rats were subjected to a training experiment for 4 consecutive days. First, the rats were placed in a water maze without any platforms for adaptive training. Specifically, the rats were allowed to swim freely in the water maze for 120 s, and then, they were taken out and cleaned to minimize the effects of factors such as grasping and water environment. After that, the navigation test was performed as follows. A transparent cylindrical escape platform with a diameter of 10 cm was placed in one of the quadrants randomly, and the surface of the platform was 2 cm from the water surface. Then, the rats facing the pool wall were randomly placed into one of the quadrants, the time of rats finding the platform (escape latency) was recorded, and the recording was stopped after 10 s of rats reaching the platform. If the platform was not found within 120 s, the rats were guided to the platform, and after 10 s, they were taken out. The test was conducted once in the morning and once in the afternoon for a total of 4 days. The escape latency, the number of crossing the intersections of platform quadrants, and the time spent in target quadrants were recorded by a computerized tracking/image analyzer system [19].

### 2.3. Novel Object Recognition Test

NORT was applied to assess cognitive dysfunction in rats based on the spontaneous tendency of rats to show more interactions with novel objects rather than familiar objects. On the first day of the adjustment phase, each rat was free to explore the open field (a 55 cm × 55 cm × 38 cm white box) without objects. During the familiarization phase on the second day, each rat was placed in the white box containing two identical objects for 5 min. On the third day, one of the familiar objects was replaced with a novel one, i.e., rats were exposed to a familiar object and a novel object to test their recognition memory. The exploration time of rats to familiar objects (TF) and novel objects (TN) was recorded during each phase, and the recognition index (RI) of finding the novel object was calculated according to the formula of RI = TN/(TF + TN) [20].

### 2.4. Fear Conditioning Test

Contextual and tone fear conditioning (CFC and TFC) tasks were set to evaluate associative emotional memory, and rats were placed in a conditioning box to receive CFC and TFC training [21]. In the CFC test, rats were placed in a conditioning box with striped walls, grid floors, and bright lights for 5 min, followed by a 20 s of sound (80 dB) stimulation. Subsequently, a 0.8 mA foot shock was given for 3 s, a total of three times; 1 min later, the rats were returned to the cage. After 24 hours, the CFC test was performed. Briefly, the rats were placed again in the conditioning box for 5 min, and in this process, they didn't suffer sound and foot shock stimulation. After 24 hours of CFC testing, TFC testing was conducted. The rats were put in a box with a semicircular white wall and flat floor for 5 min, followed by sound stimulation twice (80 dB, 20 s, interval of 1 min). By the way, during TFC testing, the box was cleaned with 30% acetic acid, and the lamp was turned off. The whole process of the above testing was videotaped for subsequent analysis, the freezing behavior of rats associated with contextual fear memory caused by spontaneous activities and adverse experiences was recorded, and the percentage of freezing behavior time was calculated using Image FZ software.

### 2.5. ELISA Assay

Hippocampal tissues were isolated from the brain tissues (50 mg) of rats in each group. After the addition of PBS buffer, the tissues were fully homogenized and then centrifuged at 12,000 r/min for 30 min at 4°C to obtain the supernatant. Subsequently, the levels of IL-1*β*, IL-6, monocyte chemoattractant protein-1 (MCP-1), brain-derived neurotrophic factor (BDNF), and nerve growth factor (NGF) in the hippocampal tissues of rats in each group were measured in accordance with the corresponding ELISA assay kit (Solarbio, China) instructions strictly.

### 2.6. Malondialdehyde Level and Glutathione Peroxidase Activity Assay

Likewise, hippocampal tissues were isolated from the brain tissues (50 mg) of rats in each group. Then, PBS buffer was added, and the tissues were homogenized thoroughly. After centrifugation at 12,000 r/min for 30 min at 4°C, the supernatant was collected. Next, the levels of malondialdehyde (MDA) and glutathione peroxidase (GSH-Px) in rat hippocampal tissues were measured under the instructions of the biochemical assay kit (Nanjing Jiancheng Bioengineering Institute, China).

### 2.7. HE Staining

Hippocampal tissues (approximately 0.5 cm around the injury site) from rats in each group were collected. Then, the tissues were fixed in 4% paraformaldehyde for 24 h and embedded in paraffin, and 6 *μ*m thick sections were made. The sections were subjected to dewaxing with xylene, dehydration with graded ethanol, hydration, and staining with HE staining kit (Solarbio, China). After dehydration with alcohol and xylene, the sections were sealed with neutral resin, and finally, histopathological changes of hippocampal tissues in rats were observed under a light microscope (Olympus, Japan), and rated images were collected.

### 2.8. Western Blot

The total protein was extracted from hippocampal tissues using RIPA lysate (Solarbio, China), followed by the detection of the concentration with a BCA kit (Solarbio, China). Next, 5 × loading buffer was added, and 20 *μ*g of the total protein was boiled. After denaturation, the protein was separated by sodium dodecyl sulfate-polyacrylamide gel electrophoresis (SDS-PAGE). Later, the protein was transferred to a PVDF membrane (Millipore, USA), and the membrane was blocked with 5% skimmed milk powder for 2 to 3 h and then incubated overnight with the primary antibodies (antiglucose-regulated protein 78 (GRP78), anti-C/-EBP homologous protein (CHOP), antitranscription factor 6 (ATF6), antitoll like receptor 4 (TLR4), anti-NF-*κ*B 65 (*p*65), anti-p-*p*65, antitumor-necrosis-factor-*α* (TNF-*α*), anti-*β*-actin; Cell Signaling Technology, USA). Therefore, the membrane was washed with TBST 3 times and incubated with the secondary antibody (ZSGB-BIO, China) for 1 h at ambient temperature. Again with TBST washing 3 times, ECL chemiluminescent reagent (Biyotime, China) was added to the membrane. Then, the protein was developed by a gel imaging system, and the images were collected. Ultimately, the gray level of the protein bands was analyzed by Image J software, and the relative protein expression was calculated by taking *β*-actin as an internal reference.

### 2.9. Data Analysis

All data were expressed as mean ± standard deviation (SD) and visualized and statistically analyzed by GraphPad Prism 9.0 software. A one-way analysis of variance was used for comparison among multiple groups. *P* < 0.05 was deemed as the criterion for the significance of difference.

## 3. Results

### 3.1. Naringin Attenuates D-Galactose-Induced Spacial Learning and Memory Dysfunction in Rats

**Figure 1:**
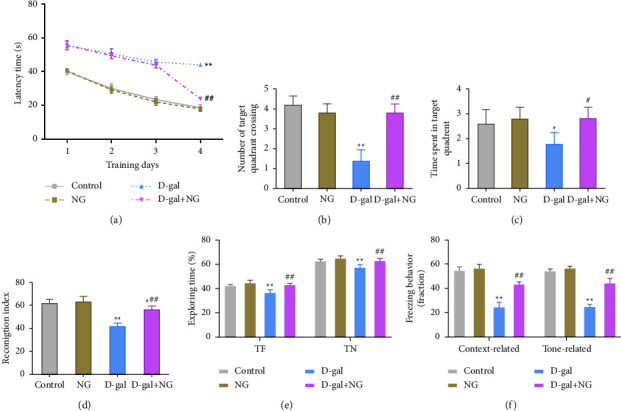
Naringin attenuates D-galactose-induced spatial learning and memory dysfunction in rats. (a)–(c): the effects of naringin on escape latency (a), the number of target quadrant crossing (b), and the time spent in the target quadrant (c) in aging rats observed through the Morris water maze test; (d)–(f): the novel object recognition test was applied to assess the effects of naringin on novel object exploration recognition index (d), percentage of time spent exploring familiar objects and novel objects (e), and percentage of time spent in contextual and context fear conditioning tests (f) in aging rats. ^*∗*^*P* < 0.05, ^*∗∗*^*P* < 0.01, vs. control; ^#^*P* < 0.05 and ^##^*P* < 0.01, vs. D-gal.

To reveal the effect of naringin on cognitive dysfunction in aging rats, we established an aging rat model with D-gal (150 mg/kg)-induced spacial learning and memory dysfunction. The results showed that, compared with the control group, rats in the D-gal group exhibited prolonged escape latency, reduced number of target quadrant crossing, and shortened time spent in the target quadrant. Besides, the recognition index (RI), the percentage of time spent exploring familiar and novel objects, and the percentage of freezing time of rats in the D-gal group were significantly lower than the control group, suggesting that the model was successful. Moreover, compared with the D-gal group, rats in the D-gal + NG group showed shortened escape latency, increased number of target quadrant crossing, and prolonged time spent in the target quadrant, as well as a significant increase in the RI, the percentage of time spent exploring familiar and novel objects, and the percentage of freezing time (Figures 1(a)–1(f)). The above outcomes presented that naringin treatment could significantly attenuate D-gal-induced spatial learning and memory dysfunction in rats.

### 3.2. Naringin Alleviates D-Galactose-Induced Pathological Damage in Rat Hippocampus

**Figure 2:**
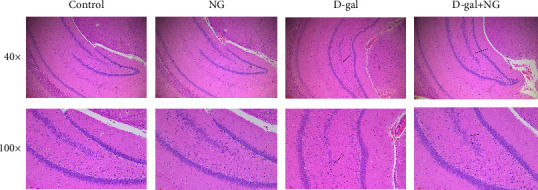
Naringin alleviates D-galactose-induced pathological damage in the rat hippocampus.

The hippocampal tissues from each group were sectioned and stained with HE to directly observe the effect of naringin on D-gal-induced hippocampal histopathology in rats. Specifically, the hippocampal tissues in the D-gal group presented severe neuron and vertebral cell damage, while in the NG group, the cellular structure of the hippocampal tissues of D-gal rats was normal, and only a few cells suffered from karyopyknosis and cytoplasmic vacuolization (Figure 2). The above findings suggested that naringin significantly alleviated D-gal-induced pathological damage in the hippocampal tissues of rats.

### 3.3. Naringin Attenuates D-Galactose-Induced Inflammatory Response and Oxidative Stress in Rat Hippocampus

**Figure 3:**
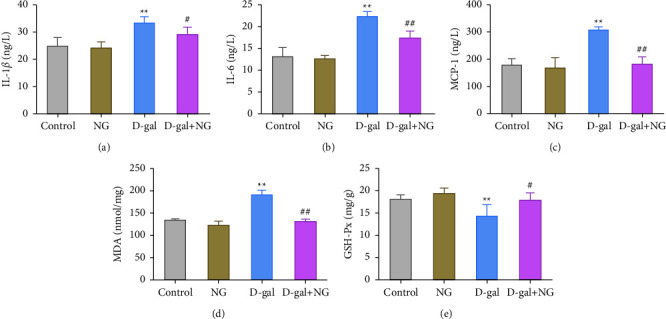
Naringin attenuates D-galactose-induced inflammatory response and oxidative stress in the rat hippocampus. (a)–(c): the levels of proinflammatory cytokines IL-1*β* (a), IL-6 (b), and MCP-1 (c) in the hippocampus of rats in each group detected by ELISA assay; (d)/(e): the levels of oxidative stress substance MDA (d) and the activity of GSH-Px (e) in the hippocampus of rats in each group detected by the biochemical kit; ^*∗∗*^*P* < 0.01, vs. control; ^#^*P* < 0.05 and ^##^*P* < 0.01, vs. D-gal.

The imbalance of inflammatory response and oxidative stress further damaged hippocampal tissues. ELISA and biochemical assays were used to measure the levels of inflammatory factors and oxidative stress substances in the hippocampal tissues of each group. As shown in Figures 3(a)–3(e), the levels of proinflammatory cytokines IL-1*β*, IL-6, and MCP-1 in the D-gal group were significantly increased compared with those in the control group, while gavage with naringin could markedly inhibit the levels of IL-1*β*, IL-6, and MCP-1 in the D-gal group (Figures 3(a)–3(c)). As for oxidative stress, the level of MDA in the D-gal group was greatly increased, and the activity of GSH-Px was declined compared with the control group. After NG treatment, the level of MDA was considerably downregulated, and the activity of GSH-Px was restored (Figures 3(d) and 3(e)). In short, naringin could alleviate D-gal-induced inflammatory response and oxidative stress in rat hippocampus.

### 3.4. Naringin Inhibits D-Galactose-Induced Endoplasmic Reticulum Stress in Rat Hippocampus

**Figure 4:**
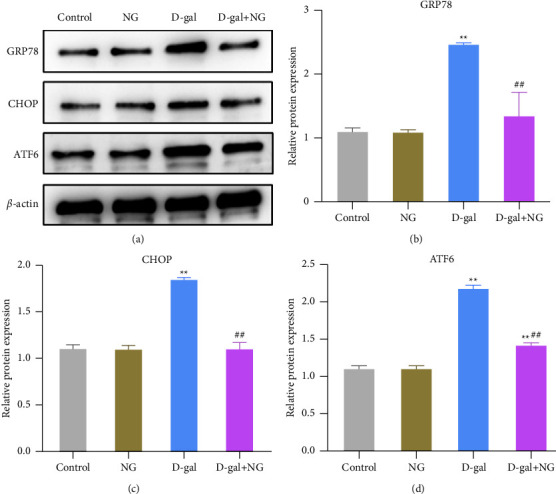
Naringin inhibits D-galactose-induced endoplasmic reticulum stress in the rat hippocampus. (a)–(d): the relative protein expression levels of ER stress-related proteins GRP78, CHOP, and ATF6 in the hippocampus of rats in each group detected by western blot; ^*∗∗*^*P* < 0.01, vs. control; ^##^*P* < 0.01, vs. D-gal.

ER stress is a marker of impaired hippocampal tissue function. In order to clarify the effect of naringin on D-gal-induced ER stress in rat hippocampal tissues, the expression levels of ER stress-related proteins (GRP78, CHOP, and ATF6) in the hippocampal tissues of rats in each group were examined. According to the experimental data, the expression levels of GRP78, CHOP, and ATF6 in the D-gal group were significantly increased while decreased in the D-gal + NG group (Figures 4(a)–4(d)). Briefly speaking, naringin was able to inhibit D-gal-induced ER stress in rat hippocampal tissues.

### 3.5. Naringin Increases the Levels of D-Galactose-Induced Neurotrophic Factors in Rat Hippocampus

**Figure 5:**
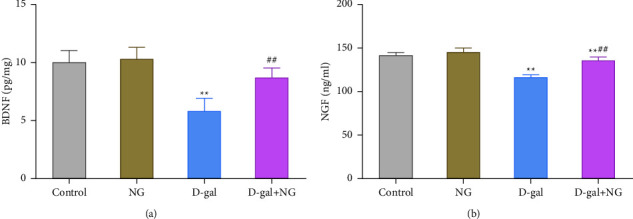
Naringin increases the levels of neurotrophic factors in the D-galactose-induced rat hippocampus. The levels of brain-derived neurotrophic factor (BDNF) (a) and nerve growth factor (NGF) (b) in the hippocampus of rats in each group were assessed by ELISA assay; ^*∗∗*^*P* < 0.01, vs. control; ^##^*P* < 0.01, vs. D-gal.

Neurotrophic factors are essential factors to maintain the normal function of hippocampal tissues. We observed that the levels of BDNF and NGF in the D-gal group were much lower than those in the control group, while after NG treatment, BDNF and NGF levels in the hippocampal tissue of D-gal rats were significantly increased (Figures 5(a) and 5(b)). Briefly, naringin could increase the levels of neurotrophic factors in D-gal-induced rat hippocampal tissues.

### 3.6. Naringin Inhibits the TLR4/NF-*κ*B Pathway in D-Galactose-Induced Rat Hippocampus

**Figure 6:**
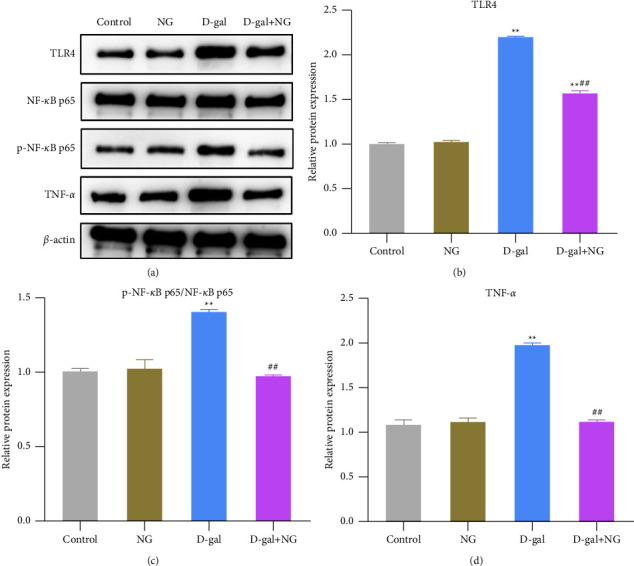
Naringin inhibits the TLR4/NF-*κ*B pathway in D-galactose-induced rat hippocampus. (a)–(d): the relative protein expression levels of TLR4/NF-*κ*B pathway-related proteins TLR4, p65 (NF-*κ*B), p-p65 (p-NF-*κ*B), and TNF-*α* in the hippocampus of rats in each group detected by western blot; ^*∗∗*^*P* < 0.01, vs. control; ^##^*P* < 0.01, vs. D-gal.

Studies reported that TLR4/NF-*κ*B signaling pathway is associated with cognitive dysfunction caused by various factors [22, 23]. Nevertheless, it is unclear whether it plays a role in alleviating D-gal-induced cognitive dysfunction by naringenin. In order to further reveal the mechanism of naringin protecting rats from D-gal-induced cognitive dysfunction, the expression of TLR4/NF-*κ*B pathway-related proteins was detected by western blot. The results disclosed that, compared with the control group, the protein expression levels of TLR4, p-NF-*κ*B p65, and TNF-*α* and the ratio of p-NF-*κ*B p65/NF-*κ*B were significantly increased in the D-gal group, while compared with the D-gal group, the D-gal + NG group exhibited decreased TLR4, p-NF-*κ*B p65, TNF-*α* protein expression levels, and p-NF-*κ*B *p*65/NF-*κ*B ratios (Figures 6(a)–6(d)). The above results indicated that D-gal could significantly activate the TLR4/NF-*κ*B pathway in rat hippocampus, while naringin treatment could inhibit the activation of the TLR4/NF-*κ*B pathway.

## 4. Discussion

The physiological process of aging involves progressive cognitive loss caused by brain function deterioration, including the decline in learning and memory skills [24]. The D-gal-induced aging model is widely used in antiaging studies due to its aging characteristics such as short life span, cognitive dysfunction, learning and memory dysfunction, and decreased immune function [25]. Moreover, conventional and quantitative D-gal injections in rats may trigger symptoms similar to natural aging [25]. It has been shown that in the D-gal-induced aging model in rats, excessive and long-term D-gal injection leads to excessive production of advanced glycosylation end-products (AGEs) and reactive oxygen species (ROS), which in turn produce intense oxidative stress and chronic inflammatory responses, thereby accelerating aging in rats [26]. Oxidative stress accumulation and chronic low-grade inflammation are considered as hallmarks of aging, which cause damage to neurons and synapses in the brain and eventually lead to impaired cognitive function [27]. Several studies have confirmed that oxidative stress factors and proinflammatory factors are accumulated gradually while antioxidant factors and anti-inflammatory factors are markedly downregulated in both hippocampal tissues and serum of aging rats with cognitive dysfunction [28]. In our study, rats were subcutaneously injected with D-gal (150 mg/kg) for the construction of the aging rat model. The behavioral test results revealed that D-gal severely impaired the learning and memory ability and hippocampal tissues of rats, promoted inflammatory response, ER stress, and oxidative stress, and reduced the levels of neurotrophic factors. The above outcomes in this study indicated that an aging rat model of cognitive dysfunction was constructed successfully.

Generally, due to good anti-inflammatory and antioxidant effect, naringin can significantly inhibit the levels of inflammatory factors and oxidative stress in hippocampal tissues [29]. Moreover, as a novel drug for the treatment of Parkinson's disease, naringin activates antiapoptotic programs by inducing the production of neurotrophic substances such as BDNF and vascular endothelial growth factor [30]. Intriguingly, in our research, naringin obviously improved the learning and memory abilities of the aging rats. Besides, naringin exerted anti-inflammatory and antioxidant effects by inhibiting the levels of inflammatory cytokines IL-1*β*, IL-6, and MCP-1 and regulating the levels or activity of oxidative stress-related substances in the hippocampus of aging rats. Furthermore, naringin could notably increase the levels of BDNF and NGF in the hippocampus of D-gal group rats. Ramalingayya et al. discovered that naringin greatly alleviated short-term episodic memory deficits in rats [31]. Jiang et al. pointed out that naringin provided neuroprotection for CCL2-induced cognitive dysfunction in aging rats by attenuating neuronal apoptosis in the hippocampus [32]. In a word, naringin has a good neuroprotective effect.

ER is a vital organelle and a crucial component of the proteostasis network. Aging-caused ER function declines result in the accumulation of the misfolded proteins in the ER cavity, thereby leading to ER stress [33]. GRP78, one of the members of the heat shock protein family in ER cavity, is a hallmark of ER stress [34]. During ER stress, GRP78 binds to unfolded proteins and activates multipartner complexes, then results in the increase of ER protein folding capacity and accumulation of unfolded or misfolded proteins, and finally induces apoptosis [34]. In prior reports, naringin exhibited its inhibition effect on ER stress. For instance, Wang et al. stated that naringin alleviated cerebral ischemia-reperfusion injury in rats by inhibiting ER stress [35]. And Albayrak et al. discovered that naringin could affect ER stress in colon cancer cells through ATF4/CHOP pathway [13]. In our paper, naringin was able to significantly reduce protein levels of GRP78, CHOP, and ATF6 in hippocampal tissues of D-gal rats. All of the above findings suggest that naringin can effectively reduce ER stress in the hippocampus of D-gal aging rats.

TLR4, as a critical receptor for both innate and adaptive immunity, can activate NF-*κ*B signaling and further induce downstream inflammatory response [36]. After activation and translocation to the nucleus, NF-*κ*B induces a series of gene expressions mediating immunity, cell adhesion, inflammation, and apoptosis [37, 38]. Previous research has proved that naringin affects neuronal apoptosis via the TLR4/NF-*κ*B pathway [39]. In our investigation, naringin could significantly downregulate the activity of the TLR4/NF-*κ*B pathway activated by D-gal. Chtourou et al. also claimed that naringin might inhibit TLR4/NF-*κ*B pathway through antioxidation and anti-inflammation [40]. However, our study involved neither the exploration of the specific mechanism of naringin regulating TLR4/NF-*κ*B nor the detection of the activity of additional signaling pathways that may be related to TLR4/NF-B. As a result, it is unclear whether naringin exerts a protective effect by virtue of other signaling pathways, and the mechanism of action of naringin also needs further exploration and validation.

## 5. Conclusion

In summary, naringin inhibits D-gal-induced inflammatory response, oxidative stress, and ER stress in rats, alleviates pathological damage, and increases the levels of neurotrophic factors in rat hippocampus, thereby alleviating cognitive dysfunction in aging rats. According to further mechanistic studies, naringin may exert its protective effect by inhibiting the activity of the TLR4/NF-*κ*B signaling pathway. In short, naringin is a beneficial option for the treatment of elderly patients with cognitive dysfunction.

## Data Availability

The data used to support the findings of this study are available from the corresponding author upon request.
